# Editorial: Radiomics and connectomics: applications to central nervous system diseases

**DOI:** 10.3389/fneur.2024.1513927

**Published:** 2024-12-11

**Authors:** Lin Wu, Fuqing Zhou, Meng Li

**Affiliations:** ^1^The First Affiliated Hospital of Nanchang University, Nanchang, Jiangxi, China; ^2^Jena University Hospital, Jena, Germany

**Keywords:** radiomics, connectomics, CNS diseases, MRI, neuroradiology

The brain, often referred to as the command center of human behavior, is a complex network of billions of synapses that transmit signals in various patterns and sequences. These intricate processes are responsible for every thought, emotion, function, and dysfunction that defines us. The field of neuroscience is rapidly evolving, with significant advancements being made in understanding the complexities of the central nervous system (CNS). Our Frontiers Research Topic, “*Radiomics and connectomics: applications to central nervous system diseases*,” showcases the application of these cutting-edge methodologies to unravel the complexities of CNS diseases.

The four accepted manuscripts in this Research Topic exemplify the power of functional and structural MRI in advancing our understanding of CNS diseases. Each study delves into different aspects, from morphometric features to functional connectivity, providing a comprehensive view of the brain's intricate workings and the impact of diseases on these processes.

The first manuscript, “*Altered cortical and subcortical morphometric features and asymmetries in the subjective cognitive decline and mild cognitive impairment*” (Yang et al.), investigates the structural changes in the brain that occur in the early stages of cognitive decline. This study contributes significantly to the identification of potential biomarkers for early diagnosis and intervention in Alzheimer's disease.

The second manuscript, “*Amygdala and cognitive impairment in cerebral small vessel disease: structural, functional, and metabolic changes*” (Cheng et al.), explores the role of the amygdala in cognitive impairment associated with cerebral small vessel disease. This manuscript also explore how new neuroimaging methods can assess amygdala changes early, laying a foundation for future comprehensive exploration of the pathogenesis of cerebral small vessel disease, offering new insights into the pathophysiology of vascular cognitive impairment.

The third manuscript, “*Hypo-connectivity of the primary somatosensory cortex in Parkinson's disease: a resting-state functional MRI study*” (Wang et al.), investigates the connectivity of the primary somatosensory cortex in Parkinson's disease. This study provides valuable insights into the sensorimotor network disruption in Parkinson's disease, contributing to the understanding of non-motor symptoms and their impact on quality of life for patients.

Finally, the fourth manuscript, “*Altered regional neural activity and functional connectivity in patients with non-communicating hydrocephalus: a resting-state functional magnetic resonance imaging study*” (Huang et al.), examines the neural activity and connectivity in patients with non-communicating hydrocephalus. The findings point to the disrupted regional neural activity and functional connectivity are altered in patients with non-communicating hydrocephalus and are correlated with cognitive impairment in cognitive impairment, advancing our understanding of the pathophysiological mechanisms underlying the association between non-communicating hydrocephalus and cognitive impairment.

In summary, the articles featured in this Research Topic showcase the transformative potential of functional and structural MRI in advancing our understanding of CNS diseases. They highlight the importance of multimodal imaging techniques in uncovering the complex neural substrates underlying cognitive impairments, and they set the stage for future research that will undoubtedly lead to improved diagnostic accuracy, more effective treatments, and ultimately, better patient outcomes. We are grateful to all the authors for their groundbreaking contributions and look forward to witnessing the continued growth and evolution of this exciting field.

